# Facing a request for assisted death - views of Finnish physicians, a mixed method study

**DOI:** 10.1186/s12910-024-01051-x

**Published:** 2024-05-03

**Authors:** Reetta P. Piili, Minna Hökkä, Jukka Vänskä, Elina Tolvanen, Pekka Louhiala, Juho T. Lehto

**Affiliations:** 1 Faculty of Medicine and Health Technology, Tampere University, Tampere, Finland; 2 Palliative Care Centre, Tampere University Hospital, Palliative Care Unit, Sädetie 6, R-building, Tampere, 33520 Finland; 3 Diaconia University of Applied Sciences, Helsinki, Finland; 4 Research Unit of Health Sciences and Technology, University of Oulu, Oulu, Finland; 5 Finnish Medical Association, Helsinki, Finland; 6 Faculty of Social Sciences, Tampere University, Tampere, Finland

**Keywords:** Assisted death, Euthanasia, Physician-assisted suicide, Physician, Ethics

## Abstract

**Background:**

Assisted death, including euthanasia and physician-assisted suicide (PAS), is under debate worldwide, and these practices are adopted in many Western countries. Physicians’ attitudes toward assisted death vary across the globe, but little is known about physicians’ actual reactions when facing a request for assisted death. There is a clear gap in evidence on how physicians act and respond to patients’ requests for assisted death in countries where these actions are not legal.

**Methods:**

A survey including statements concerning euthanasia and PAS and an open question about their actions when facing a request for assisted death was sent to all Finnish physicians. Quantitative data are presented as numbers and percentages. Statistical significance was tested by using the Pearson chi-square test, when appropriate. The qualitative analysis was performed by using an inductive content analysis approach, where categories emerge from the data.

**Results:**

Altogether, 6889 physicians or medical students answered the survey, yielding a response rate of 26%. One-third of participants agreed or partly agreed that they could assist a patient in a suicide. The majority (69%) of the participants fully or partly agreed that euthanasia should only be accepted due to difficult physical symptoms, while 12% fully or partly agreed that life turning into a burden should be an acceptable reason for euthanasia. Of the participants, 16% had faced a request for euthanasia or PAS, and 3033 answers from 2565 respondents were achieved to the open questions concerning their actions regarding the request and ethical aspects of assisted death. In the qualitative analysis, six main categories, including 22 subcategories, were formed regarding the phenomenon of how physicians act when facing this request. The six main categories were as follows: providing an alternative to the request, enabling care and support, ignoring the request, giving a reasoned refusal, complying with the request, and seeing the request as a possibility.

**Conclusions:**

Finnish physicians’ actions regarding the requests for assisted death, and attitudes toward euthanasia and PAS vary substantially. Open discussion, education, and recommendations concerning a request for assisted death and ethics around it are also highly needed in countries where euthanasia and PAS are not legal.

**Supplementary Information:**

The online version contains supplementary material available at 10.1186/s12910-024-01051-x.

## Background

Practices of assisted death (euthanasia and assisted suicide) have been adopted in several Western countries across the globe. The Netherlands, Belgium, Luxembourg, Canada, the states of Victoria and Western Australia in Australia, New Zealand, and Spain have legalized euthanasia [1, 2]. Assisted suicide is legal in Switzerland and eight states in the USA (Maine, New Jersey, Oregon, Washington, Montana (court ruling), Vermont, Colorado, Hawaii, and California) and the District of Columbia [1, 2]. In Switzerland, assisted suicide is also available for Swiss nonresidents [1, 2]. In Columbia, there is a court ruling that physicians are not to be prosecuted for euthanasia or physician-assisted suicide (PAS), and Germany decriminalized assisted suicide in 2021 [1, 2]. Euthanasia is illegal in Finland. Assistance in suicide is not mentioned in Finnish law and the potential consequences to a physician performing PAS are not known as this has never been tested in a court of law. Nevertheless, neither euthanasia nor PAS has been legalized or practiced in Finland. Discussions about the legalization of practices of assisted death are ongoing in many countries around the globe, including Finland.

According to previous studies, physicians support euthanasia and PAS less than the public [3], but the variation in attitudes toward assisted death among physicians in different countries is large. In Finland, the number of physicians fully agreeing with the statement “Euthanasia should be legalized in Finland” has increased from 5% in 1993 to 25% in 2020 [4]. On the other hand, the number of physicians fully disagreeing with this statement also increased from 30 to 34% between 1993 and 2020 [4]. The number of Finnish physicians fully disagreeing with the statement ”A physician should be punished for assisting in a suicide” has increased from 14 to 39% between 1993 and 2020 [4]. In Sweden, acceptance of PAS has risen among physicians from 35% in 2007 to 47% in 2020 [5].

Concerns about the impact of euthanasia and assisted suicide requests on physicians have been raised [6, 7]. Physicians have been described as having evolved different feelings after hastening the death of a patient, such as satisfaction or relief, and feelings such as loneliness, tension, and discomfort [6, 8–11]. The process of assisted death was also described as burdensome, both emotionally and bureaucratically [6, 8–11]. Another study from Canada revealed that medical assistance in dying had enriched the capacity for caring and altered the relationship with patients and families [12]. A recent narrative systematic review revealed mixed feelings among healthcare professionals, such as anxiety, frustration, and moral or emotional distress, as well as feelings of success in alleviating symptoms [13]. There is also evidence that refusal of the request for assisted death impacts physicians, as physicians have reported having mixed feelings afterward [7]. According to a qualitative study from Canada, the most expressed reasons for not participating in medical aid in dying were the emotional burden related to this act and the fear of psychological repercussions [7].

Most of the studies concerning physicians’ responses and actions when facing a request for assisted death have been qualitative interviews with a small number of participants or questionnaires from countries where assisted death is legal [14–21]. Only a few studies have explored physicians’ actions in countries where assisted death is illegal, and these studies have been based on questionnaires without a real opportunity to explain one’s actions and the reasoning behind them [20, 21]. There is a clear gap of evidence on how physicians act and respond to patients’ requests for assisted death in a larger scope in countries where these actions are not a legal possibility.

The aims of this study were to assess Finnish physicians’ views on assisted death and to describe their actions when facing a request for assisted death.

## Methods

A mixed method study design was used where both quantitative and qualitative analysis approaches were launched in parallel. The mixed methods approach was chosen to get a thorough understanding of the phenomenon in interest [22]. The quantitative methods used were descriptive statistics [23]. In addition, the study applied a descriptive qualitative approach with an inductive content analysis method [24]. The intention of the qualitative analysis was to present a comprehensive summary of the phenomenon of interest, without claiming any methodological roots [25].

### Participants

An email survey concerning assisted death was sent to all Finnish physicians and medical students with permission to practice medicine, who are members of the Finnish Medical Association, and whose email addresses were available (*n* = 26,740).

### Questionnaire

The survey questionnaire included several statements concerning euthanasia and PAS. In the questionnaire, PAS was defined as follows: a physician deliberately helping a person commit suicide by giving drugs to the person to take them by him/herself on this person’s voluntary and competent request. Euthanasia was defined as follows: a physician deliberately killing a patient by administering drugs at the patient’s voluntary and competent request. The legal status of these actions was also mentioned in the questionnaire; Euthanasia is covered under criminal law in Finland [26]. Because suicide is not considered a criminal act, assistance in suicide is not regarded as a criminal act. However, healthcare professionals have a special obligation to protect the patients of whom they take care; thus, it can be assumed that the act of a physician will not remain unpunished. The participants were asked to express their agreement on statements concerning assisted death on a Likert scale: fully agree, partly agree, partly disagree, fully disagree or I cannot say. Furthermore, participants were asked if they had faced a request for euthanasia or assistance in suicide by a patient or a patient´s relative. If the participant answered yes, an open question followed, “Would you describe the situation and your actions shortly?” Another open question was also included: “If there is anything else you would like to share concerning euthanasia or physician-assisted suicide, please tell us”. Additionally, some background information, such as age, gender, and self-reported experience in the care of dying patients (yes or no) and for how long (not at all/less than 5 years/5–10 years/more than 10 years), was asked. The questionnaire is available as an additional file.

Some of the results from this survey have been previously published [4].

### Ethical considerations

This survey was conducted through the member registry of the Finnish Medical Association. The association has permission to send questionnaires to its members if they have not declined this. Responding was anonymous, and participation was voluntary. Participants gave their consent by voluntarily answering the questionnaire. The research data did not include any personally identifiable data. According to Finnish legislation, ethics approval is not needed in this type of questionnaire study [27]. This study was conducted according to national laws, regulations, and the Declaration of Helsinki.

### Data analysis

The answers of the respondents are described with numbers and proportions. When assessing relations between background factors and the answers concerning the statements “I could assist a patient in a suicide”, “A physician should be able to assist a patient in a suicide”, “Legislation should confirm that a physician assisting a patient in a suicide will not be punished”, “If euthanasia would be legalized in Finland, acceptable reasons for euthanasia should only be difficult physical symptoms (e.g., pain and dyspnea) in the end stage of a disease” and “If euthanasia would be legalized in Finland, life turning into an unbearable burden, should also be accepted as a reason for euthanasia”, the Likert scale was converted to two options: fully/partly agree and fully/partly disagree/I can’t say (Table 3). These two-scale answers and background factors were tested using the Pearson chi-square test. Two-sided *p*-values less than 0.05 were considered statistically significant. Data analysis was performed using IBM SPSS Statistics for Windows, Version 27.0 (IBM Corporation, 2020).

The qualitative data consisted of answers to open questions: “If you answered yes [the responder had faced a request for euthanasia or assistance in suicide by a patient or a patient´s relative], would you describe the situation and your actions shortly?” and “If there is anything else you would like to share concerning euthanasia or physician-assisted suicide, please tell us”. The qualitative analysis was performed by using an inductive content analysis approach, where categories emerge from the data. As recommended in guidelines [24, 28], no theoretical framework was used as a starting point, and the manifest contents were analyzed. The units of analysis consisted of words, sentences, or phrases that constructed a meaning. The analysis followed four phases: (1) transcribing the data verbatim from the questionnaires to a Microsoft Word template and familiarizing with the data; (2) reducing and coding the data that were relevant to the study aim; (3) grouping the reduced expressions based on similarities; and (4) forming subcategories and categories of the data. An example of the coding process is shown in the additional file 2. The reduced expressions were formed by two of the authors (blinded) after which the other authors went through the results. Once consensus was achieved, one of the authors then performed the categorizations and abstraction of the data, which was again critically assessed by all the other authors. Data saturation was achieved during the analysis, which indicates that the data were sufficient [29]. After the reduction phase, the data consisted of 761 reduced expressions which refers to the richness of the data. The frequencies (f) of the reduced expressions are presented to show the noteworthiness of each category in relation to the entirety.

## Results

Altogether 6889 physicians or medical students answered the survey, yielding a response rate of 26%. The characteristics of the participants are shown in Table 1. Most of the participants were female (59%), and 61% of all participants were at least 45 years old. Approximately one out of four participants were retired. Approximately two-fifths were involved in taking care of dying patients at the time of answering the survey, mostly with less than five years of experience. On the other hand, one-third of the participants had more than ten years of experience in the care of dying patients. Of the participants, 16% had faced a request for euthanasia or a wish for assistance in suicide from a patient or a patient’s relative.

**Table 1:** Characteristics of the participants

	*n*	(%)
Number of participants	6889	
Response rate		(26)
Female	4020	(59)
Age distribution		
< 35 y	1437	(21)
35–44 y	1220	(18)
45–54 y	1131	(16)
55–64 y	1292	(19)
≥ 65 y	1798	(26)
Specialty in a full-time job		
Operative	1408	(21)
Conservative	1645	(24)
Diagnostic	426	(6)
Psychiatric	566	(8)
General medicine, occupational medicine, and other fields	1611	(23)
Not specialized	1218	(18)
Current working status		
Working	4656	(68)
Student	400	(6)
Retired	1626	(24)
Out of work due to another reason	191	(3)
Currently taking care of dying patients		
Yes	2859	(42)
No	3953	(57)
Amount of experience in the care of dying patients		
Not at all	1113	(16)
< 5 y	2778	(41)
5–10 y	912	(13)
> 10 y	2050	(30)
Patient or patient’s relative having asked for euthanasia or PAS		
Yes	1068	(16)
No	5821	(85)

The answers to the statements concerning euthanasia and PAS are reported in Table 2. One-third of participants agreed or partly agreed that they could assist a patient in a suicide. The statement “A physician should be able to assist a patient in a suicide” divided the participants in half. In addition, most (60%) of the participants agreed, fully or partly, that legislation should confirm that a physician assisting a patient in a suicide will not be punished. The majority (69%) of the participants fully or partly agreed that euthanasia should only be accepted due to difficult physical symptoms, while 12% fully or partly agreed that life turning into a burden should be an acceptable reason for euthanasia. Associations between background factors and statements are presented in Table 3. Males, younger respondents, and respondents who had faced a request for assisted death more often agreed that they could assist a patient in suicide. Males and respondents who had faced a request for an assisted death were more in favor of euthanasia also being granted on the basis of life turning into an unbearable burden. Most experienced physicians and physicians participating in the care of the dying agreed least that physicians should be able to assist in suicide.

**Table 2:** Agreement with statements

Statement, *n* (%)		Fully agree			Partly agree			Partly disagree			Fully disagree			I cannot say	
I could assist a patient in a suicide		897	(13)		1361	(20)		595	(9)		3027	(44)		984	(14)
A physician should be able to assist a patient in a suicide		1153	(17)		2025	(30)		1048	(15)		465	(32)		465	(7)
Legislation should confirm that a physician assisting a patient in a suicide will not be punished		2400	(35)		1706	(25)		895	(13)		1136	(17)		722	(11)
If euthanasia would be legalized in Finland, acceptable reasons for euthanasia should only be difficult physical symptoms (e.g., pain and dyspnea) in the end stage of a disease		2630	(38)		2140	(31)		844	(12)		416	(6)		813	(12)
If euthanasia would be legalized in Finland, life turning into an unbearable burden, should also be accepted as a reason for euthanasia		183	(3)		638	(9)		1195	(17)		4411	(64)		431	(6)

**Table 3:** Background factors of the participants in a relation to statements

	I could assist a patient in a suicide		*P*-value*	A physician should be able to assist a patient in a suicide		*P*-value*	Legislation should confirm that a physician assisting a patient in a suicide will not be punished		*P*-value*	If euthanasia would be legalized in Finland, acceptable reasons for euthanasia should only be difficult physical symptoms (e.g., pain and dyspnea) in the end stage of a disease		*P*-value*	If euthanasia would be legalized in Finland, life turning into an unbearable burden should also be accepted as a reason for euthanasia		*P*-value*
	Fully/Partly agree, n (%)			Fully/Partly agree, n (%)			Fully/Partly agree, n (%)			Fully/Partly agree, n (%)			Fully/Partly agree, n (%)		
Gender			*< 0.001*			*< 0.001*			0.033			0.071			*< 0.001*
Female	1159	(29)		1751	(44)		2352	(59)		2830	(71)		361	(9)	
Male	1070	(39)		1388	(50)		1704	(61)		1905	(69)		443	(16)	
Age			*< 0.001*			*< 0.001*			0.435			*0.009*			*0.004*
< 35 y	595	(42)		721	(50)		856	(60)		994	(69)		189	(13)	
35–44 y	407	(33)		552	(45)		723	(59)		825	(68)		120	(10)	
45–54 y	352	(31)		531	(47)		690	(61)		752	(67)		128	(11)	
55–64 y	329	(26)		531	(41)		745	(58)		904	(71)		137	(11)	
> 65 y	572	(32)		837	(47)		1086	(61)		1291	(73)		246	(14)	
Specialty in a full-time job			*< 0.001*			*< 0.001*			*< 0.001*			*< 0.001*			*< 0.001*
Operative	520	(37)		711	(51)		921	(66)		1007	(72)		183	(13)	
Conservative	432	(26)		671	(41)		925	(57)		1178	(72)		165	(10)	
Diagnostic	178	(42)		241	(57)		273	(64)		300	(71)		66	(16)	
Psychiatric	145	(26)		245	(44)		320	(57)		375	(67)		56	(10)	
General medicine, occupational medicine, and other fields	495	(31)		696	(43)		925	(58)		1137	(71)		178	(11)	
Not specialized	483	(40)		605	(50)		733	(60)		765	(63)		171	(14)	
Participates in the care of dying patients			0.373			*< 0.001*			*< 0.001*			*0.017*			0.061
Yes	923	(32)		1204	(42)		1624	(57)		1938	(68)		317	(11)	
No	1312	(33)		1942	(49)		2440	(62)		2783	(71)		496	(13)	
Amount of experience			*< 0.001*			*< 0.001*			*< 0.001*			0.284			0.107
Not at all	367	(33)		593	(54)		734	(66)		782	(71)		156	(14)	
< 5 y	1007	(36)		1339	(48)		1660	(60)		1897	(69)		325	(12)	
5–10 y	311	(34)		410	(45)		535	(59)		652	(72)		111	(12)	
> 10 y	559	(27)		819	(40)		1152	(57)		1418	(70)		228	(11)	
Patient or patient’s relative having asked for euthanasia or PAS			*< 0.001*			*< 0.001*			*< 0.001*			0.397			*< 0.001*
Yes	470	(44)		569	(54)		698	(66)		730	(69)		173	(16)	
No	1788	(31)		2609	(45)		3408	(60)		4040	(70)		648	(11)	

* Pearson Chi-Square

### Physicians’ actions when facing a request for assisted death

Altogether, 3033 answers from 2565 respondents were included in the inductive analysis.

**Fig. 1:**
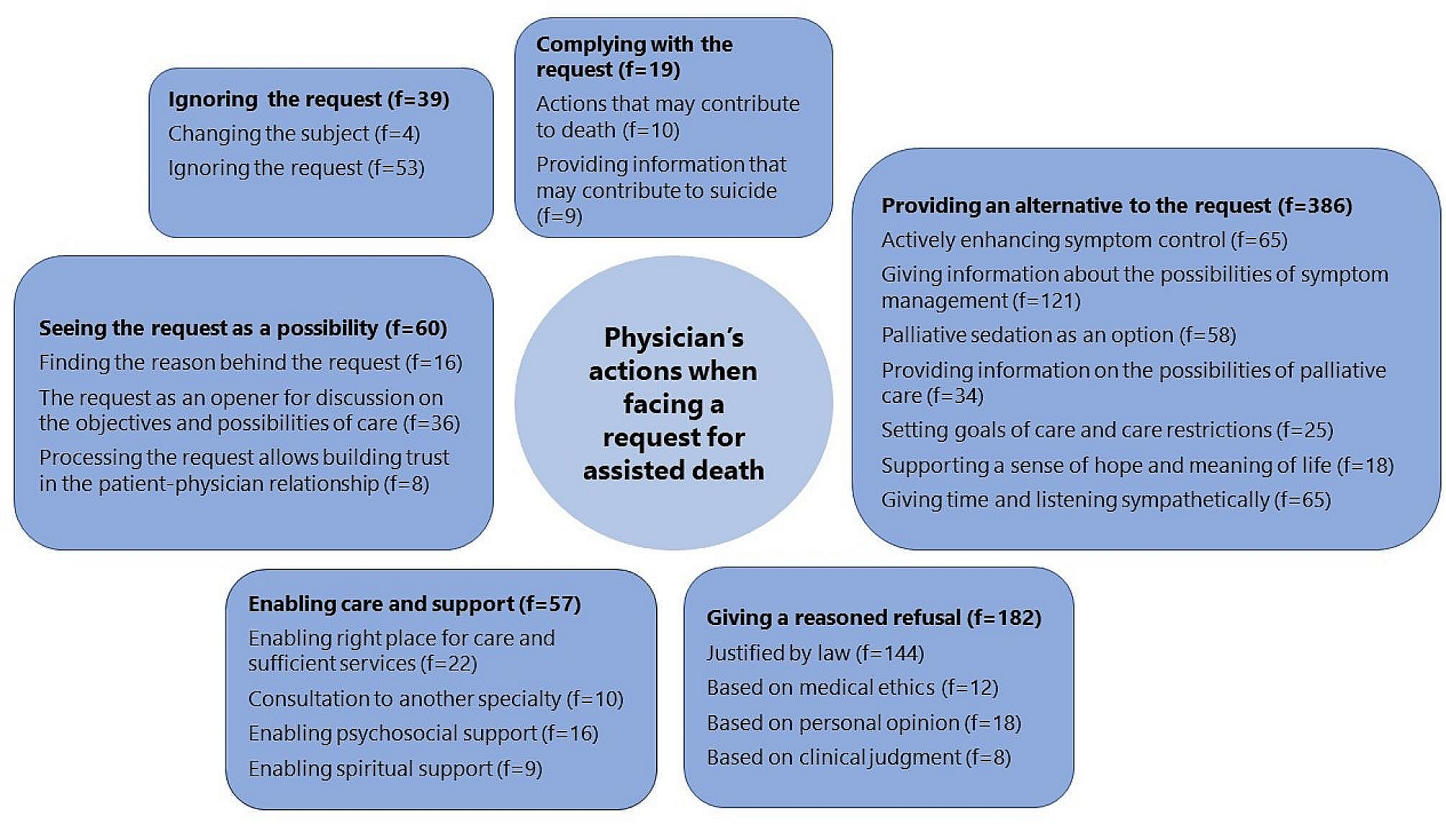
Physicians’ actions when facing a request for assisted death

Six main categories emerged from the data, namely, providing an alternative to the request, enabling care and support, ignoring the request, giving a reasoned refusal, complying with the request, and seeing the request as a possibility. The six main and 22 subcategories are shown in Fig. 1 and described in detail below.

### Providing an alternative to the request

Many physicians described an active approach when faced with a request for assisted death. Through their actions, they aimed to offer alternatives for their patient. They tried to improve the care of patients’ symptoms when possible and necessary. Some of the physicians described they aimed to reduce patient concerns by providing information about the possibilities of symptom control in the expected suffering. One way to give an alternative was to tell about the possibilities of palliative sedation to relieve suffering. Sometimes the criteria for sedation were already met, and the patient was sedated. A few physicians described that they explained the possibilities of palliative care. Setting the goals of care and making care restrictions together with the patient was also a viable alternative.

*I asked the patient what symptoms he had. I told about the possibilities for symptom relief, especially pain relief.1214Q11*.

*I have also talked about the possibility of palliative sedation to ease the suffering of the last moments of life. 662Q1*.

Physicians also responded that giving time and discussing with the patient and loved ones had been a valuable solution for the situation. They emphasized the importance of supporting a sense of hope and meaning in life for the patient and family.

*A calm conversation often eases this burden (anxiety of loved ones) as well. 3169Q11*

*In these situations, I have tried to find ways to alleviate the patient’s often very long-term suffering by other means, i.e., to help them cope with hopelessness, … the experience of meaninglessness. 4268Q11*

### Enabling care and support

In this category, the physicians themselves were not the ones providing the care and support but acted as facilitators so that the patients and closest ones received the help they needed. The physicians addressed the possibility of enabling sufficient services and a care environment as a way to respond to the request. They also mentioned the importance of psychosocial and spiritual care for the patient and family. One way to act in the situation was also to consult a senior, another specialty, or a specialist palliative care unit.

*At the appointment, we can focus on concrete solutions to alleviate patient suffering, such as psychosocial support. 938Q11*.

*I referred him to a neurologist who knows him. The neurologist made a referral to a specialist palliative care unit, and the patient gave up his plans for euthanasia. 2117Q11*.

### Ignoring the request

Some of the physicians described that they didn’t want to face the request, but it was easier to try to avoid the conversation. For some of the physicians, ignoring the request totally was a way to act when meeting the request for assisted death. This took the form of ignoring the request by changing the subject without giving the patient the possibility to return to the subject. Physicians also described ignoring the request without further discussion as a way of dealing with the situation.

*I avoided the question and changed the subject of the conversation. 401Q11*

*I rejected the request. 906Q11*

### Giving a reasoned refusal

In this category, physicians refused the request for assisted death, but they provided a reason for the patient. There were several different reasons that the physicians described. Many physicians mentioned that they discussed with the patient that they would refuse the request. They justified their refusal to the patient based on the law, medical ethics, or their own personal values. Some physicians told the patient that, based on medical assessment, euthanasia would not be possible in the patient’s situation even if euthanasia were legal because the criteria for euthanasia would probably not be met.

*I told the patient my role and values are to promote and support life. 639Q11*

*I refused justifying it based on medical ethics to sustain life. 3387Q11*

### Complying with the request

Some of the physicians also had mixed thoughts about how to respond to the request. Some physicians raised concerns about whether their actions in end-of-life situations had enabled hastened death. A few expressed presumptions that their actions had facilitated patient death or suicide. A few also raised the issue of having facilitated suicide by providing information on the possibility of assisted suicide in another country.

*In repeated requests, I said that the drugs are such that an overdose will kill, but I did not say directly how. 183Q11*

*I directed the patient to contact a Swiss clinic. 3829Q11*

### Seeing the request as a possibility

In this category, the physicians stated that the request was seen as an opportunity that paved the way for further discussions. Some of the physicians saw the request as a possibility to improve the patient-physician relationship and achieve better care, e.g., through discussion on the underlying causes for the request. The physicians also often opened discussions about the goals of care and care options with the patient after the request, which they saw more as a discussion opener than an actual request for death.

*Discussions often open many perspectives on the care of the patient and the reasons behind the anxiety and the request. 1314Q11*.

*Usually, these discussions have been good, and the patient has understood that it is not possible. The discussions have also increased confidence in symptom management and the physician. 1667Q11*.

## Discussion

This study reveals new and more in-depth knowledge about physicians’ actions when facing a request for assisted death in a country where euthanasia and PAS are not legal. Physicians have adopted various ways to deal with a request for assisted death, as they also have different attitudes toward euthanasia and PAS.

In our study, only 13% fully agreed with the statement “I could assist a patient in a suicide”. In previous studies, willingness to perform euthanasia or PAS among physicians has varied widely from 2 to 16% in Germany, 8% in the United States, and 30% in Italy [30–32]. A recent questionnaire study from Sweden showed that 33% of respondents were willing to prescribe the drugs needed to perform assisted suicide in 2020 [5]. On the other hand, in the Netherlands and Belgium, where assisted death has been legal for decades, 86% and 81% of physicians could imagine a circumstance in which they might participate in the practice of euthanasia or PAS [3].

Most participants agreed that euthanasia should be accepted only in difficult physical symptoms in the end stage of a disease. Difficult physical symptoms have been one of the reasons for euthanasia or PAS in many countries [30, 31]. Others include for example, loss of function, dependency or loss of independence, deterioration, loss of dignity, and hopelessness [33, 34]. In a study from Oregon, as many as 57% of patients reported loss of independence as a reason for requests for PAS [33]. In our study, only 3% fully agreed with the statement “If euthanasia would be legalized in Finland, life turning into an unbearable burden, should also be accepted as a reason for euthanasia”. Males and physicians who had faced these requests agreed fully or partly agreed more often (16% in both groups) with this in our study. This question aimed to ask whether the responder thinks that euthanasia with unbearable suffering without unbearable physical symptoms would be an acceptable reason for euthanasia. In many countries ‘unbearable suffering’ is a criterion for euthanasia, but only when it occurs together with a disease. The complexity of unbearable suffering is reflected by the ongoing debates regarding whether euthanasia and assisted suicide should be permitted for psychiatric disorders. In some countries, including Belgium and the Netherlands, it is legal to perform assisted death based on psychiatric disorders [30]. A systematic review from 2020 showed that articles providing ethical reasoning and opinions in favor of or against assisted death based on psychiatric disorders were evenly distributed [35].

In our study, male and young physicians thought more often that they could assist in a suicide, which probably reflects the overall more positive attitude in these groups regarding practicing PAS and euthanasia [4, 5, 32]. In addition, if a physician had faced a request for assisted death, they were more likely to have positive attitudes toward euthanasia and PAS. No previous studies were found to support this finding.

The amount of experience in the care of dying patients was associated with less agreement with assisting in a suicide and with the general view that physicians should not assist in a suicide. In a study from Germany, physicians with special qualifications in palliative care were more reluctant to hasten a patient’s death through euthanasia or PAS [30], which is in line with our findings. It is also known from previous studies, that physicians with the most experience with end-of-life care and palliative care have been most reluctant toward euthanasia and PAS [36, 37], and this finding is again repeated in this study. The reasons behind this have not been profoundly studied. However, it can be argued that knowledge and experience with palliative and end-of-life care can provide more options to take care of the patient. It might also be better understood among physicians with experience in dealing with end-of-life issues that a patient’s wish to hasten death does not always imply a genuine wish to die [38–40]. It might be a result of overwhelming physical, psychological, social, and existential suffering, all of which have an impact on the patient’s sense of self, dignity, and meaning in life [38–40].

This study showed that physicians face the request for assisted death in their everyday practice even if it is not legal in Finland. However, the requests were not very common, as only 16% of participants reported having been asked for euthanasia or assistance in suicide. In a study from Sweden, half of the physicians who participated in that study had heard their patients expressing a wish to die, but only a few had asked for euthanasia or assisted suicide [21]. In an older study from England, as many as 45% of physicians who responded to a questionnaire, reported having been asked for euthanasia [20].

Physicians reported diverse ways of responding to the request and actions they took when meeting the request for assisted death. There is relatively little research about requests for assisted death when it is not legal. It is known that the patient´s wish for euthanasia could persist for at least one year despite the wish being declined [41]. Additionally, a small qualitative study from the Netherlands found that the wish to die is not abandoned, although the request has been refused [42]. Based on these results, ongoing discussions and suggestions for practice are needed when these requests are faced in countries where assisted death is not a legal option or when the request is rejected in the countries allowing assisted death.

In the results of the qualitative data of this study, many physicians expressed that knowledge of the possibility of palliative sedation at the end of life could comfort patients frightened of suffering at the end of life when assisted death is not a legal possibility. There is only a limited amount of knowledge on the relationship between assisted death and palliative sedation. In a study from Switzerland, continuous deep sedation was not considered an alternative to assisted suicide, but temporary or intermittent sedation was sometimes introduced in response to a request for assisted suicide [43].

In this study, the request was also sometimes seen as a possibility to enhance the care and find the underlying reasons for the death wish. The results also showed that physicians were seeking alternatives to alleviate suffering, including improving symptom management, maintaining hope and a sense of meaning in life, and providing an appropriate place of care and adequate support for the patient. In a Swedish study, some respondents answered that a request for euthanasia might express wishes for the alleviation of symptoms or wider communication: after talking, these requests disappear [21].

Ignoring the request was one way of dealing with the request in our study. However, ignoring the request for assisted death could indicate that the reasons behind the death wish are ignored [41]. Therefore, it could be stated that refusal without further discussion or support is not the optimal way to act when meeting the request for assisted death.

Some physicians responded to comply or partly comply with the request, e.g. describing drugs or recommending contacting a Swiss clinic. In Scandinavia, euthanasia or assistance in suicide is very rarely reported by physicians [21, 44–46]. This is understandable, as euthanasia is under the criminal code in all Scandinavian countries.

Some fears of whether one´s actions had hastened the patient´s death were reported in this study. Hastening a patient’s death or a fear of doing so when alleviating severe symptoms or withdrawal of treatment, is by far more difficult and ethically challenging question, and is sometimes confused with euthanasia or PAS [47]. A large multinational study performed in 2005 found that there was general approval for alleviating symptoms with possible life-shortening treatment among physicians [48]. Similar findings were discovered in a European study from six different countries, where 57–95% of physicians were willing to intensify the drug therapy to alleviate pain and/or other symptoms, although they considered that there was a probability or certainty that this would shorten a patient’s life [49].

Some of the physicians expressed mixed feelings about what would be the right way to act when facing a request for assisted death. This calls for recommendations or guidelines on how to act when meeting the request. Only a few recommendations have been published where practical guidance on how to respond to the request for assisted death is provided and some of them apply merely in countries where assisted death is possible to practice [50–52]. The most important recommendation for health care professionals in these articles is to try to understand the meaning behind the request and to be able to face the difficult emotions the request evokes both in a patient and in the professionals [50–52].

### Strengths and limitations

The study population is a large and representative sample of Finnish physicians [53], although the response rate was rather low, and possible nonresponse bias must be taken into account. The sampling, data collection, and analysis process were reported in detail, which increases the reliability of the study. The sample included physicians with different backgrounds, such as different specialties and amounts of experience. Therefore, it can be assumed that the study population gave a large and versatile view of physicians’ attitudes toward assisted death and how they act when facing a request for assisted death. Furthermore, dependability was strengthened by presenting the figure of all the categories (Fig. 1), and authenticity was strengthened by providing authentic citations of the data. It should also be noted that the researchers constantly discussed the analysis throughout the study. Confirmability was strengthened by focusing on the manifest content during the analysis when it can be assumed that the results would represent the views of the physicians [28].

There are also several limitations in this study. Nonresponse bias might have affected the results, but the number of respondents was, however, substantial. Furthermore, there was no possibility to return the qualitative findings to the physicians for comments or corrections [24]. The questionnaire used in this study is the same that has been used in a series of surveys and to maintain comparability, the questions and statements were similar to the previous ones [4]. There are clear differences in the ethical and practical issues between euthanasia and PAS, but in the open-ended question and some other parts of our results, these two methods of assisted death were combined. This should be taken into account when interpreting our results. However, the request for assisted death may be presented without a specific definition of PAS or euthanasia and both are unlegalized in Finland. Thus, we do believe the answers of the respondents reflect the overall views of the Finnish physicians concerning assisted death and experiences when facing the request for this.

## Conclusions

Our findings can be considered unique, and they bring new and relevant knowledge to the difficult subject of assisted death. When facing a request for assisted death, Finnish physicians’ actions vary substantially. Some physicians use the request as a way to guide therapeutic options and support offered to the patients, while others practically ignore the request. Most of them are not willing to assist a patient in a suicide, but attitudes toward assisted death are divided. Open discussion, education, and recommendations about facing a request for assisted death and ethics around it are also highly needed in countries where assisted death is not a legal possibility.

### Electronic supplementary material

Below is the link to the electronic supplementary material.

**Additional file 1**: Questionnaire.

**Additional file 2**: Example of coding.

## Data Availability

The datasets generated and/or analyzed during the current study are not publicly available due to the current data policy of the Finnish Medical Association but are available from the corresponding author on reasonable request.

## Abbreviations

PAS: physician-assisted suicide
