# Supplementary material for: Facing a request for assisted death - views of Finnish physicians, a mixed method study
Source: BMC Med Ethics. 2024 May 3;25:50. doi: 10.1186/s12910-024-01051-x (PMC11067268; doi:10.1186/s12910-024-01051-x)
Supplement: Supplementary file 1 — Additional file 1: Questionnaire. [file 12910_2024_1051_MOESM1_ESM.docx]

Questionnaire 2020

Euthanasia and physicians-assisted death in 2020- a questionnaire for physicians

* question is mandatory and determines next questions for you

1) Gender

female male other I don’t want to say

2) Age group

under 35 years 35-44 years 45-54 years 55-64-years over 64 years

3) Specialty in your full-time job

operative conservative diagnostic psychiatric general medicine, occupational medicine, and other fields (forensic medicine, health care medicine, sports medicine)

4) Your current working status

working student retired out of work due to another reason

5) Where do you work at the moment?

primary health care specialized care occupational health service Other, clarify_____

6) Do you take part in the care of dying patients in your current work position?

yes no

7) How much experience do you have in caring for dying patients?

not at all less than 5 years 5-10 years more than 10 years

**Questions concerning physician-assisted death**

*Physician-assisted death is defined as a physician deliberately helping a person to commit suicide by giving drugs to the person to take them by him/herself by this person’s voluntary and competent request.*

*Currently, assisting in a suicide is not considered a criminal act according to criminal law because suicide is not regarded as a criminal act. However, health care professionals have a special obligation to protect patients of whom they take care; thus, it can be assumed that the act of a physician will not remain unpunished.*

What is your opinion about the following statements concerning physician-assisted death?

A physician should be punished for assisting in a suicide.

| Fully agree | Partly agree | Partly disagree | Fully disagree | I can’t say |
| --- | --- | --- | --- | --- |

A physician should be able to assist a patient in a suicide

| Fully agree | Partly agree | Partly disagree | Fully disagree | I can’t say |
| --- | --- | --- | --- | --- |

Legislation should confirm that a physician assisting a patient in a suicide will not be punished

| Fully agree | Partly agree | Partly disagree | Fully disagree | I can’t say |
| --- | --- | --- | --- | --- |

I would assist a patient in a suicide

| Fully agree | Partly agree | Partly disagree | Fully disagree | I can’t say |
| --- | --- | --- | --- | --- |

**Questions concerning euthanasia**

*Euthanasia is defined as a physician deliberately killing a patient by administering drugs by the patient’s voluntary and competent request.*

*Euthanasia is covered under criminal law in Finland and can be punished as manslaughter.*

What is your opinion about the following statements concerning euthanasia?

Euthanasia should be legalized in Finland.

| Fully agree | Partly agree | Partly disagree | Fully disagree | I can’t say |
| --- | --- | --- | --- | --- |

A practice similar to that in the Netherlands and Belgium, where euthanasia is legal under precise conditions, should be adopted.

| Fully agree | Partly agree | Partly disagree | Fully disagree | I can’t say |
| --- | --- | --- | --- | --- |

With adequate terminal care and pain control, there is no need for euthanasia.

| Fully agree | Partly agree | Partly disagree | Fully disagree | I can’t say |
| --- | --- | --- | --- | --- |

Accepting euthanasia would harm the doctor–patient relationship in general.

| Fully agree | Partly agree | Partly disagree | Fully disagree | I can’t say |
| --- | --- | --- | --- | --- |

If euthanasia is legalized in Finland, the acceptable reasons for euthanasia should be only difficult physical symptoms (e.g., pain and dyspnea) in the end stage of a disease.

| Fully agree | Partly agree | Partly disagree | Fully disagree | I can’t say |
| --- | --- | --- | --- | --- |

If euthanasia is legalized in Finland, life turning into an unbearable burden should also be accepted as a reason for euthanasia.

| Fully agree | Partly agree | Partly disagree | Fully disagree | I can’t say |
| --- | --- | --- | --- | --- |

Accepting euthanasia would benefit the doctor–patient relationship in general.

| Fully agree | Partly agree | Partly disagree | Fully disagree | I can’t say |
| --- | --- | --- | --- | --- |

*Has a patient or a patient’s relative asked for euthanasia or physician assisted death from you?

Yes No

If you answered yes, please describe the situation and your actions briefly:

____________________________________________________________________________

**Current stance of the Finnish Medical Association**

*The Finnish Medical Association objects to the legalization of euthanasia. The Finnish Medical Association also objects that physicians would be obliged to perform procedures that aim at hastening a patient’s death (physician-assisted death).*

Should the Finnish Medical Association change its stance on euthanasia and physician-assisted death?

No

Yes, the Finnish Medical Association should change its stance on euthanasia.

Yes, the Finnish Medical Association should change its stance on physician-assisted death.

I can’t say

What should be the stance of the Finnish Medical Association on euthanasia?

­_______________________________________________________________________

What should be the stance of the Finnish Medical Association on physician-assisted death?

­_______________________________________________________________________

If there is anything else you would like to share with the Finnish Medical Association concerning euthanasia or physician-assisted death, please tell us:

_______________________________________________________________________
