# Supplementary material for: Facing a request for assisted death - views of Finnish physicians, a mixed method study
Source: BMC Med Ethics. 2024 May 3;25:50. doi: 10.1186/s12910-024-01051-x (PMC11067268; doi:10.1186/s12910-024-01051-x)
Supplement: Supplementary file 2 — Additional file 2: Example of coding. [file 12910_2024_1051_MOESM2_ESM.docx]

Additional file 2. An example of the coding procedure how the subcategory ‘Finding the reason behind the request’ was produced inductively.

| Examples of the original data | Examples of codes (reduced expressions) | Subcategory |
| --- | --- | --- |
| 3520Q11 I've interpreted these as openings for discussion, wanting to make sure that help is definitely available and many patients want to hear that there are ways to alleviate symptoms behind the request. | 3520Q11 The request is opening for discussion to make sure to tell there are help to treat symptoms behind the request. |  |
| 662Q11 I have explored the issues that have led to the desire for euthanasia. | 662Q11 Exploring issues that led to the desire for euthanasia |  |
| 183Q11 In these situations I have discussed with the patient why they want euthanasia. | 183Q11 Discussing why they want euthanasia. |  |
| 4560Q11 Was distressed about his life for reasons that I felt were remediable. There were pains and relationship problems.  The request to kill was a plea in my opinion | 4560Q11 The request of euthanasia was a plea |  |
| 4549Q11 I have generally not commented on the request itself, but have taken it as an opening for discussion and then discussed e.g. life and the patient's wishes for his/her care more fully. | 4549Q11 Taking the request as an opening for discussion of life and patient’s wishes |  |
| 4279Q11During the discussion at the time of the request for euthanasia, the patient is given the promise to 1) take his/her concerns seriously (reason behind the request of euthanasia), | 4279Q11 at the time of the request for euthanasia, the patient is given the promise to take his/her concerns seriously (reason behind the request of euthanasia), | Finding the reason behind the request |
| 4278Q11 After making a request for euthanasia, consciously invested more than usual in active listening, in hearing the reason behind the request. | 4278Q11 consciously invested more in active listening, in hearing the reason behind the request. |  |
| 3344Q11 Discussed with the patient about their fears and reasons and how they can be addressed | 3344Q11 Discussing with the patient about their fears and reasons |  |
| 3000Q11 focused on the reasons for the request and what could be done about it, also allowing time for discussion about the situation. | 3000Q11 focusing on the reasons for the request and what could be done about it |  |
| 2942Q11 I have asked what the fear is and tried to explain how to manage the symptoms or issues behind the fear. | 2942Q11 Asking what the fear is and trying to manage the symptoms or issues behind the fear. |  |
| 1314Q11 Discussions often open up many perspectives on the patient's care and the reasons behind the anxiety and the request. | 1314Q11 Discussions open up perspectives on the patient's care and the reasons behind the anxiety and the request. |  |
| 2015Q11 The request opens an "easy" channel to discuss difficult feelings and experiences related to the illness. | 2015Q11 The request opens an "easy" channel to discuss difficult feelings and experiences |  |
| 286Q11 In these situations I have discussed with the patient why they wish to die.. | 286Q11 discussed with the patient why they wish to die |  |
| 536Q11 I tried to have a good interaction with the patient and relatives and tried to find the reason why the patient wished to die. | 536Q11 tried to find the reason why the patient wished to die. |  |

f, number of codes included in the subcategory.
